# The correlation between the use of ondansetron and mortality in sepsis associated encephalopathy patients: a retrospective ICU cohort study

**DOI:** 10.3389/fphar.2025.1712328

**Published:** 2025-11-10

**Authors:** Shuo Yang, Yi-Qu Wei, Ya-Zhou Liu, Xiao-Lin Wang, Jin-Xia Gao

**Affiliations:** 1 Department of Anesthesiology, Dalian Medical University Affiliated Second Hospital, Dalian, China; 2 Department of Anesthesiology, Dandong Central Hospital, Dandong, China; 3 Department of Critical Care Medicine, Dalian Medical University, Dalian, China; 4 Department of Orthopedic Medicine, Dalian Medical University, Dalian, China

**Keywords:** ondansetron (OND), sepsis associated encephalopathy, sepsi, mortality, QT prolongation

## Abstract

**Background:**

Sepsis-associated encephalopathy (SAE) is a severe complication with high mortality. The effect of ondansetron (OND) on the outcomes of SAE patients remains unclear.

**Methods:**

Using the Intensive Care IV Medical Information Mart (MIMIC-IV) database, we identified 7,829 SAE patients, comprising an OND group (n = 3,954) and a non-OND group (n = 3,875). Propensity score matching (PSM) was employed to generate 3,066 pairs of matches in a 1:1 ratio. The primary outcomes encompassed the 30-day, 90-day, 180-day, and 360-day mortality rates. The secondary outcomes included the duration of ICU stay, the duration of mechanical ventilation, and the incidence of QT interval prolongation. Survival analysis was conducted using Cox proportional hazards regression and Kaplan-Meier curves. Sensitivity analyses, including E-value assessment and a landmark analysis at 5 days to address immortal time bias, were performed. Subgroup analysis was applied to investigate potential differences in the effect of OND treatment on clinical outcomes among various subgroups.

**Result:**

Following PSM, the baseline characteristics were well-balanced between the cohorts. The group receiving OND demonstrated significantly lower mortality rates at 30 days (HR = 0.64, 95% CI [0.56–0.73], *p*<0.001), 90 days (HR = 0.75, 95% CI [0.66–0.84], *p*<0.001), 180 days (HR = 0.78, 95% CI [0.69–0.88], *p*<0.001), and 360 days (HR = 0.76, 95% CI [0.67–0.85], *p*<0.001) compared with the non-OND group. The landmark sensitivity analysis confirmed the robustness of this survival benefit (p < 0.001). Kaplan-Meier analysis confirmed a significant survival advantage for OND-treated patients with SAE. After matching, the OND group was associated with significantly shorter durations of ICU stay and mechanical ventilation compared with the non-OND group; however, the incidence of QT interval prolongation did not differ significantly between the two groups. Subgroup analysis indicated that adult patients younger than 65 years may derive greater survival benefit from OND treatment.

**Conclusion:**

In SAE patients, OND use is associated with significantly lower short- and long-term mortality, suggesting its potential as an adjunct therapy. However, further prospective randomized controlled trials are warranted to validate these results.

## Introduction

SAE represents a grave complication of sepsis, presenting significant short - term risks to patients in hospitals and ICUs. The severity of SAE is directly correlated with an elevated risk of death; the mortality rate among patients with severe SAE can reach up to 70% (Mazeraud et al., 2020; Shulyatnikova and Verkhratsky, 2020). The clinical manifestations of SAE encompass delirium, cognitive impairment, and, in severe cases, coma. Severe SAE may result in prolonged hospitalization, persistent neurological sequelae, and a diminished quality of life for survivors, thereby imposing a substantial burden on both individuals and society. Since SAE patients often lack definitive evidence of central nervous system (CNS) infections, identifying factors that influence SAE mortality is crucial. This could improve patient prognosis and alleviate the healthcare burden. Currently, the proposed mechanisms of SAE include neuroinflammation (Bourhy et al., 2022; Becher et al., 2017), blood - brain barrier dysfunction, impaired cerebral microcirculation (Orhun et al., 2020), and oxidative stress (Krzyzaniak et al., 2023). Consequently, a pivotal treatment approach for SAE involves controlling neuroinflammation and oxidative stress (Bourhy et al., 2022; Becher et al., 2017; Orhun et al., 2020; Krzyzaniak et al., 2023; Sonneville et al., 2025; Shime et al., 2025).

Sepsis patients frequently experience severe nausea and vomiting attributable to systemic inflammatory response syndrome (SIRS), metabolic disturbances, and medication side effects. Ondansetron, a potent 5-HT3 receptor antagonist, mitigates these symptoms by acting on both the vomiting center and the gastrointestinal tract, thereby enhancing patient comfort and reducing the risk of complications such as aspiration pneumonia (Wilde and Markham, 1996). Beyond its antiemetic properties, OND may also confer anti-inflammatory benefits. Animal studies show that 5-HT3 receptor antagonists can attenuate the release of inflammatory factors (e.g., TNF-α and IL-6) and ameliorate neuroinflammatory responses (Barnes et al., 1989). Furthermore, OND is neuroprotective; it inhibits 5-HT3 receptors to reduce glutamate release and oxidative stress, shielding neurons from excitotoxic damage (Holbrook et al., 2009). Recent clinical studies have suggested a potential benefit of OND treatment in critically ill populations, including patients with sepsis, traumatic brain injury (TBI), COVID-19, and acute kidney injury following cardiac surgery (Xu et al., 2024; Liu et al., 2025; Wang et al., 2024). However, the specific impact of OND on the prognosis of SAE patients remains unclear. Given the central role of neuroinflammation and oxidative stress in SAE pathogenesis, we hypothesize that OND may confer a beneficial effect by modulating these pathways.

The primary aim of this study is to investigate the association between OND administration and mortality in SAE patients. Utilizing the MIMIC-IV database, we will evaluate this association by comparing 30-day, 90-day, 180-day, and 360-day mortality rates. This research seeks to identify a potential novel therapeutic strategy for SAE in the intensive care unit, with the ultimate goal of improving prognostic outcomes.

## Methods

### Data source

The relevant data of SAE patients in this retrospective cohort study were obtained from the Medical Information MIMIC-IV database. The MIMIC-IV database includes confirmed clinical records of critically ill patients admitted to the Bethel Israel Deaconess Medical Center (BIDMC) ICU between 2008 and 2022, including demographic data, vital signs, laboratory test results, and diagnoses. Author Yang Shuo successfully completed the Collaborative Institution Training Program (CITI) exam and obtained permission to access the database (record ID: 65462239). The use of this database has been approved by the Central Institutional Review Board of the Massachusetts Institute of Technology and Beth Israel Deaconess Medical Center, without the need for informed consent.

### Study population

**FIGURE 1:**
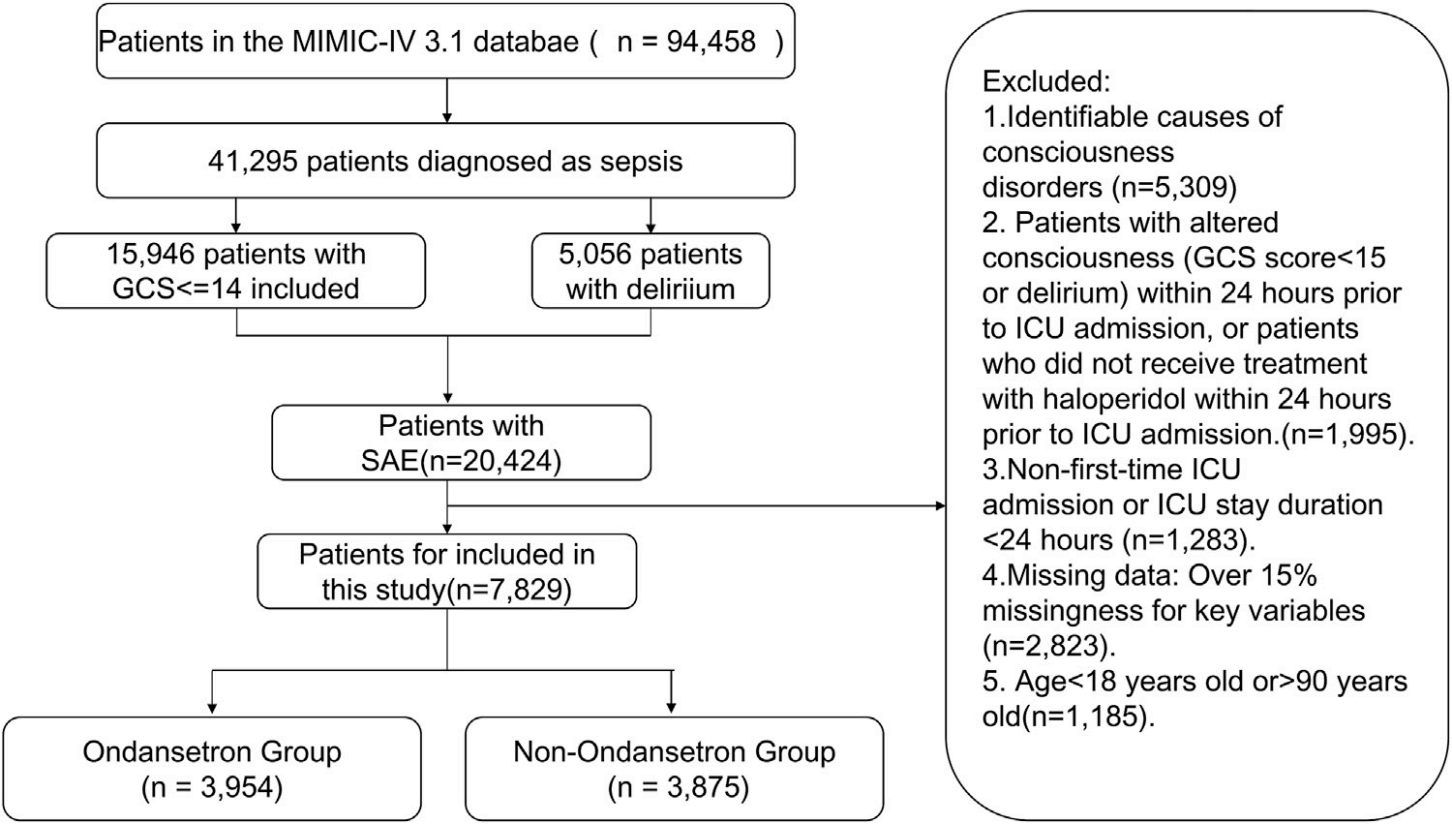
The flowchart for the selection of SAE patients.

The ICU admission records of patients were initially determined from the MIMIC-IV 3.1 database. The diagnosis of sepsis must meet the Sepsis 3.0 criteria, which requires recording or suspecting infection, and at least adding 2 points to the SOFA score (Zhang et al., 2019). Within 24 h of hospitalization in the ICU, a GCS score of ≤14 for sepsis patients indicates that changes in consciousness were assessed before administering analgesics, sedatives, or tracheal intubation. To diagnose delirium, the RASS score must first reach −3 or higher, and the ICU Confusion Assessment Method (CAM-ICU) must be used to evaluate the patient. CAM-ICU assessment includes four key features: (1) acute exacerbation of changes in mental state or fluctuations in disease progression; (2) Lack of concentration; (3) Confused thinking; (4) Changes in the level of consciousness. If a patient exhibits (1) and (2) features, as well as (3) or (4) features, within 24 h of hospitalization in the ICU, the diagnosis is delirium (i.e., CAM-ICU positive). Both groups of patients mentioned above are considered potential cases of SAE. If the patient meets the following criteria, they will be included in the study: (1) patients aged between 18 and 90 years old; (2) Patients who are admitted to the ICU for the first time and stay in the ICU for more than 24 h; (3) Patients with data missing no more than 15%. The exclusion criteria are as follows: (1) identifiable causes of consciousness disorders, including primary neurological disorders (such as cerebral infarction, cerebral hemorrhage, traumatic brain injury, central nervous system infection, epilepsy, and brain tumors); Pre existing neurological and psychiatric complications, such as mental disorders, dementia, and a long-term history of alcohol or drug abuse; Metabolic encephalopathy, including hepatic encephalopathy, hypertensive encephalopathy, and uremic encephalopathy; Severe electrolyte or blood glucose abnormalities, defined as serum sodium levels<120 mmol/L or>150 mmol/L, or blood glucose levels<54 mg/dL or>180 mg/dL; Severe respiratory acidosis, defined as PaCO _2_ ≥ 80 mmHg; (2) Patients who experience changes in consciousness (GCS score<15 or delirium) or have not received treatment with haloperidol within 24 h prior to admission to the ICU. After applying exclusion criteria, the final study population included 7,829 patients. These patients were divided into two groups based on their use of OND during their ICU stay. The OND group included 3,954 patients, while the non OND group included 3,875 patients. The patient selection process is shown in Figure 1.

### Data collection

Demographic data, including age and sex, were collected. Complications, such as hypertension, diabetes, coronary heart disease, cardiogenic shock, chronic respiratory failure, and chronic liver disease, were recorded. Vital signs comprised heart rate, mean arterial pressure, respiratory rate, and body temperature. Laboratory parameters included hemoglobin, anion gap, and serum creatinine levels. Clinical indicators involved the Charlson Comorbidity Index, the SOFA score for quantifying organ dysfunction (Ranstam and Cook, 2017), and the Simplified Acute Physiology Score (SAPS) II for predicting in-hospital mortality. Exposure factors of interest were the administration of dexmedetomidine, midazolam, and vasopressin. Data were retrieved from the Medical Information MIMIC-IV database using Structured Query Language (SQL). The MIMIC-IV database integrates de-identified electronic medical records, hospital department files, and supplementary information gathered through structured telephone interviews with patients, family members, or healthcare providers.

### Outcome

The primary outcomes of this study were mortality rates at 30 days, 90 days, 180 days, and 360 days. Secondary outcomes included ICU stay, mechanical ventilation duration, and incidence of QT interval prolongation, defined as ICD code (42682, I4581).

### Statistical analysis

The normality of continuous variables was evaluated using the Kolmogorov - Smirnov test. Variables with a normal distribution are reported as the mean ± standard deviation, whereas those with a non - normal distribution are presented as the median (interquartile range, IQR). To analyze inter - group differences, Student’s *t* - test was employed for variables with a normal distribution, and the Mann - Whitney U test was used for non - normally distributed variables. Categorical variables are expressed as frequencies (percentages) and were compared using either the chi - square test or Fisher’s exact test. PSM was carried out at a 1:1 ratio, utilizing nearest - neighbor matching without substitution (caliper width: 0.02) to balance baseline covariates. The standardized mean difference (SMD) was used to quantify the balance of clinical variables, with an SMD of less than 0.1 indicating sufficient balance (Liang et al., 2021). Cox proportional hazards regression was applied to assess the relationship between OND treatment and 30 - day, 90 - day, 180 - day, and 360 - day mortality rates, with adjustments made for confounding factors. The Kaplan - Meier survival curve was employed to compare the mortality rates between the OND - exposed and non - exposed cohorts (Ranstam and Cook, 2017). E - value analysis was performed to evaluate the potential impact of unmeasured or unknown confounding factors, aiming to determine the degree of confounding required to nullify the observed association between OND administration and mortality risk (Baxter et al., 2022). In addition, subgroup analysis was conducted to investigate the relationship between OND use and 30 days mortality rates in different subgroups. To address potential immortal time bias arising from the definition of OND exposure during the ICU stay, a landmark sensitivity analysis was performed. This analysis was restricted to patients who survived at least 5 days after ICU admission, and the association between OND treatment and mortality was re-evaluated in this cohort. Data extraction was conducted using Navicat Premium 17 (version 17.0.4), and statistical analyses were performed in R (version 4.4.1), Stata (version 18.0), and GraphPad Prism (version 10.1.2).

## Results

### Baseline characteristics

**FIGURE 2:**
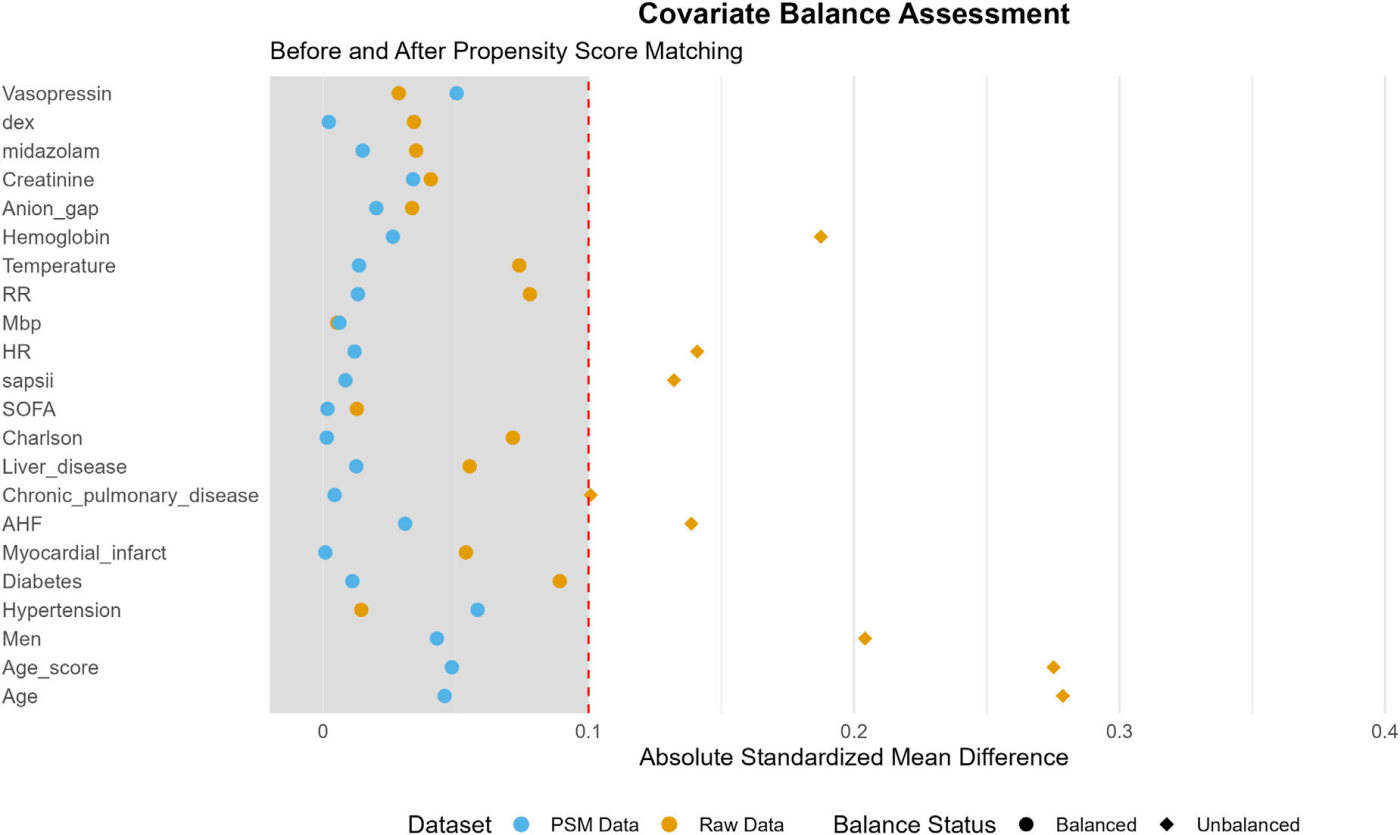
Standardized mean difference (SMD) of variables before and after propensity score matching (PSM).

This study encompassed 7,829 eligible patients, with 3,875 patients assigned to the non-OND group and 3,954 patients to the OND group. The present study is a retrospective investigation, and its findings are potentially subject to confounding factors. Following PSM, a total of 6,132 patients were incorporated into the analysis, and the influence of confounding factors was substantially mitigated. Table 1 delineates the majority of the baseline characteristics of the two patient groups both before and after PSM. In the original patient population, the clinical scores assessed by the SOFA, Oxford Acute Disease Severity Scale (OASIS), Acute Physiology Score III (APSIII), and SAPSII were lower in the OND group as compared to the non-OND group. After PSM, there was no statistically significant disparity in baseline characteristics between the two groups, with most p-values surpassing 0.05. Table 2 shows the association between ond use and the primary and secondary outcomes of all SAE patients. Figure 2 illustrates the comparison of baseline characteristics between the groups before and after PSM, with a standardized mean difference (SMD) < 0.1 for all characteristics.

**TABLE 1:** Baseline characteristics of the selected patients before and after propensity score matching.

Variables	Before PSM				After PSM			
	Total (n = 7,829)	Non-ondansetrn (n = 3,875)	Ondansetrn (n = 3,954)	P value	Total (n = 6,132)	Non-ondansetrn (n = 3,066)	Ondansetrn (n = 3,066)	P value
Demographic data								
Age Mean ± SD	66.4 ± 15.0	68.5 ± 14.4	64.4 ± 15.3	<0.001	66.7 ± 14.5	67.1 ± 14.6	66.4 ± 14.4	0.073
Men,n (%)	4,445 (56.8%)	2,397 (61.9%)	2048 (51.8%)	<0.001	3,527 (57.5%)	1796 (58.6%)	1731 (56.5%)	0.093
Comorbidities at ICU admission, n (%)								
Hypertension	4,152 (53.0%)	2041 (52.7%)	2,111 (53.4%)	0.524	3,271 (53.3%)	1,591 (51.9%)	1,680 (54.8%)	0.023
Diabetes	2,598 (33.2%)	1,368 (35.3%)	1,230 (31.1%)	<0.001	2082 (34.0%)	1,049 (34.2%)	1,033 (33.7%)	0.666
Myocardial infarct	1,435 (18.3%)	751 (19.4%)	684 (17.3%)	0.017	1,123 (18.3%)	562 (18.3%)	561 (18.3%)	0.974
AHF	2,678 (34.2%)	1,454 (37.5%)	1,224 (31.0%)	<0.001	2,101 (34.3%)	1,073 (35.0%)	1,028 (33.5%)	0.226
Chronic pulmonary disease	2,203 (28.1%)	1,179 (30.4%)	1,024 (25.9%)	<0.001	1746 (28.5%)	876 (28.6%)	870 (28.4%)	0.865
Liver disease	1,229 (15.7%)	569 (14.7%)	660 (16.7%)	0.015	972 (15.9%)	493 (16.1%)	479 (15.6%)	0.624
Vital signs								
HR Mean ± SD	88.7 ± 16.5	87.5 ± 16.3	89.8 ± 16.5	<0.001	88.7 ± 16.4	88.6 ± 16.3	88.8 ± 16.5	0.642
Mbp Mean ± SD	76.7 ± 10.1	76.7 ± 10.0	76.8 ± 10.3	0.815	76.7 ± 10.1	76.6 ± 10.0	76.7 ± 10.3	0.810
RR Mean ± SD	20.1 ± 4.1	20.3 ± 4.1	19.9 ± 4.1	<0.001	20.1 ± 4.1	20.1 ± 4.1	20.1 ± 4.1	0.607
Temperature Mean ± SD	36.9 ± 0.5	36.9 ± 0.6	36.9 ± 0.5	0.001	36.9 ± 0.5	36.9 ± 0.5	36.9 ± 0.5	0.597
Laboratory parameters								
Hemoglobin Mean ± SD	9.6 ± 2.2	9.8 ± 2.2	9.4 ± 2.2	<0.001	9.6 ± 2.2	9.7 ± 2.2	9.6 ± 2.2	0.304
Anion gap Mean ± SD	16.7 ± 5.1	16.8 ± 5.2	16.7 ± 5.1	0.138	16.8 ± 5.1	16.8 ± 5.2	16.7 ± 5.1	0.433
Creatinine Mean ± SD	1.4 ± 1.3	1.5 ± 1.3	1.4 ± 1.3	0.073	1.5 ± 1.3	1.5 ± 1.3	1.5 ± 1.2	0.184
Clinical indices								
Charlson M (Q1, Q3)	5.0 (3.0–8.0)	6.0 (4.0–8.0)	5.0 (3.0–8.0)	<0.001	5.0 (3.0–8.0)	5.0 (3.0–8.0)	5.0 (3.0–8.0)	0.988
SAPSII M (Q1, Q3)	55.0 (41.0–73.0)	57.0 (42.0–75.0)	54.0 (40.0–71.0)	<0.001	55.0 (41.0–72.0)	55.0 (41.0–72.0)	55.0 (41.0–72.0)	0.791
SOFA M (Q1, Q3)	3.0 (2.0–5.0)	3.0 (2.0–5.0)	3.0 (2.0,5.0)	0.001	3.0 (2.0–5.0)	3.0 (2.0–5.0)	3.0 (2.0–5.0)	0.249
Age score M (Q1, Q3)	2.0 (1.0,3.0)	3.0 (2.0,3.0)	2.0 (1.0,3.0)	<0.001	2.0 (1.0–3.0)	2.0 (1.0–3.0)	2.0 (1.0–3.0)	0.033
Exposure factors								
Dexmedetomidine, n (%)	811 (10.4%)	381 (9.9%)	430 (10.9%)	0.008	612 (10.0%)	305 (9.9%)	307 (10.0%)	0.932
Midazolam, n (%)	971 (12.4%)	458 (11.8%)	513 (12.9%)	<0.001	751 (12.2%)	383 (12.5%)	368 (12.0%)	0.559
Vasopressin, n (%)	1,065 (13.6%)	508 (13.1%)	557 (14.1%)	0.207	802 (13.1%)	375 (12.2%)	427 (13.9%)	0.049

Abbreviations:AHF, acute heart failure;HR, heart rate; Mbp, mean blood pressure; RR, respiratory rate; Charlson, Charlson Comorbidity Index.

Data are expressed as means or n (%). Differences between groups were analyzed using Student's t-test or Mann-Whitney U test.

**TABLE 2:** The primary, secondary and composite outcomes of the ondansetron group and non-ondansetron group before and after propensity score matching.

Variables	Before PSM				After PSM			
	Total (n = 7829)	Non-ondansetrn (n = 3875)	Ondansetrn (n = 3954)	P value	Total (n=6132)	Non-ondansetrn (n = 3066)	Ondansetrn (n = 3066)	P value
Primary outcome								
30-mortality, n (%)	1758 (22.5%)	1026 (26.5%)	732 (18.5%)	<0.001	1368 (22.3%)	776 (25.3%)	592 (19.3%)	<0.001
90-mortality, n (%)	1729 (22.1%)	1353 (34.9%)	1124 (28.4%)	<0.001	1932 (31.5%)	1038 (33.9%)	894 (29.2%)	<0.001
180-mortality, n (%)	2830 (36.1%)	1522 (39.3%)	1308 (33.1%)	<0.001	2209 (36.0%)	1170 (38.2%)	1039 (33.9%)	<0.001
360-mortality, n (%)	3173 (40.5%)	1701 (43.9%)	1472 (37.2%)	<0.001	2477 (40.4%)	1315 (42.9%)	1162 (37.9%)	<0.001
Secondary outcomes								
Icu stays M (Q1, Q3)	3.9(2.5-7.3)	4.2(2.9-8.0)	3.5(2.2-6.6)	<0.001	3.9(2.5-7.3)	4.2(2.9-8.0)	3.5(2.2-6.6)	<0.001
Ventilation M (Q1, Q3)	59.6(31.3-119.7)	64.2(35.0-129.0.)	54.9(28.5-112.4.)	<0.001	59.0(31.1-119.8)	62.2(32.7-128.0)	56.0(29.5-114.3)	0.014
QT Prolongation, n (%)	156 (2.0%)	73 (1.8%)	85 (2.1%)	0.332	123 (2.0%)	60 (2.0%)	63 (2.1%)	0.856

### Survival analysis

**FIGURE 3:**
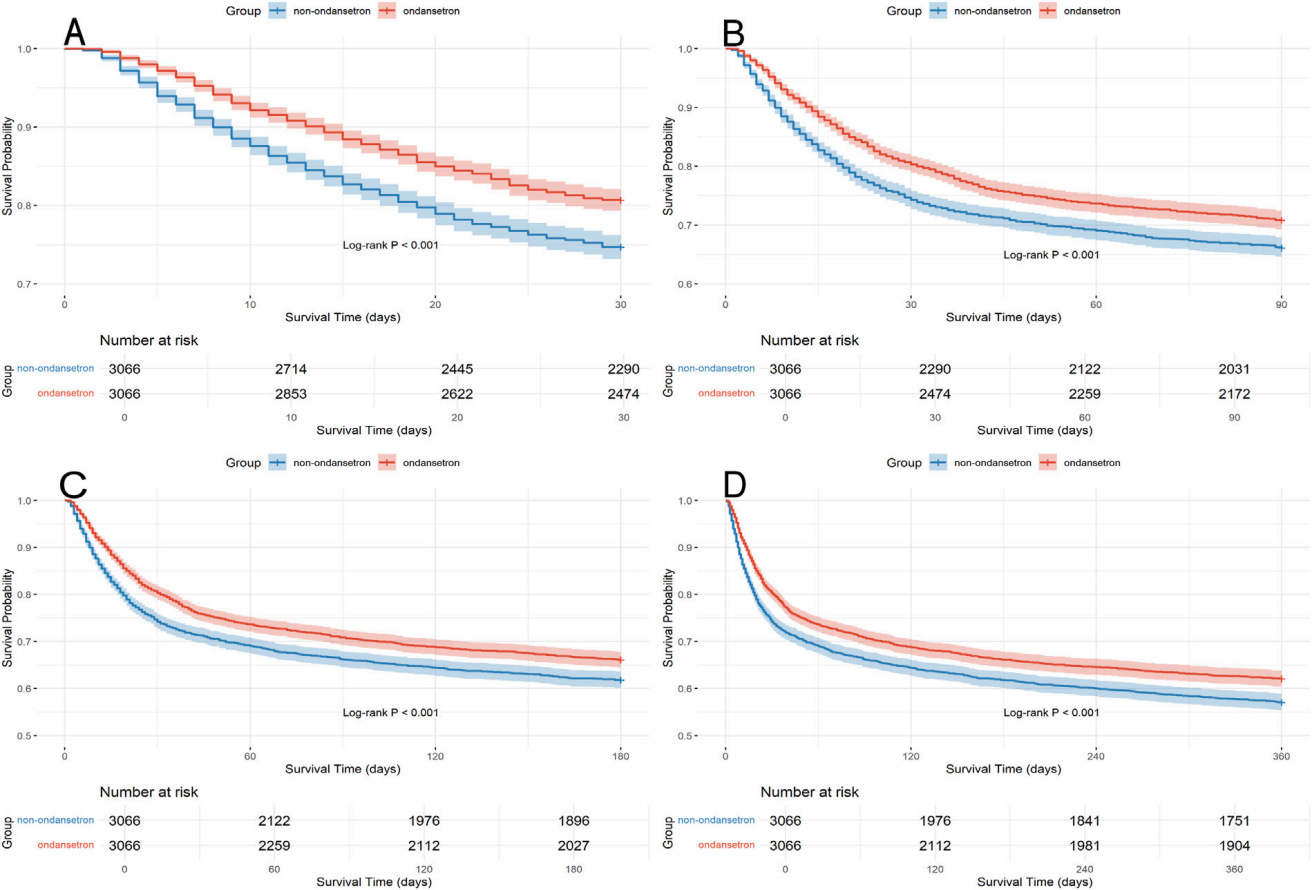
Kaplan Meier survival curves for the ondansetron group and non ondansetron group: **(A)** 30 days survival curve after PSM, **(B)** 90 days survival curve after PSM, **(C)** 180 days survival curve after PSM, **(D)** 360 days survival curve after PSM.

The Kaplan-Meier survival curve graphically compares the mortality risk between the two patient cohorts. Survival time was extracted for each patient from the database. As shown in Figure 3, OND-treated septic patients demonstrated a significantly higher probability of survival at 30, 90, 180, and 360 days compared to the non-OND group. The log-rank test yielded a p-value of <0.001, indicating a statistically significant difference in survival between the groups.

### Association between ondansetron exposure and mortality risk: Multivariate cox regression analysis

As presented in Table 3, We performed a multivariate Cox regression analysis within the PSM cohort to investigate the relationship between OND treatment and 30 - day, 90 - day, 180 - day, and 360 - day mortality rates. In the unadjusted Model 1, the hazard ratios (HRs) for 30 - day, 90 - day, 180 - day, and 365 - day mortality were 0.71 (95% confidence interval [CI]: 0.63–0.80), 0.80 (95% CI: 0.72–0.90), 0.83 (95% CI: 0.75–0.92), and 0.81 (95% CI: 0.73–0.90), respectively. Subsequently, in Model 2, we adjusted for heart rate (HR), mean blood pressure (MBP), respiratory rate (RR), and body temperature. The trends observed in Model 2 remained consistent with those in Model 1. In Model 3, we further adjusted for heart rate, mean blood pressure, respiratory rate, body temperature, age, sex, hypertension, diabetes, myocardial infarction, acute heart failure (AHF), chronic lung disease, liver disease, hemoglobin, anion gap, creatinine, Charlson comorbidity index, SAPSII, SOFA, age score, and the use of dexmedetomidine, midazolam, and vasopressin. Notably, after comprehensive adjustment, the HRs for mortality at 30 days, 90 days, 180 days, and 365 days were 0.64 (95% CI: 0.56–0.73), 0.75 (95% CI: 0.66–0.84), 0.78 (95% CI: 0.69–0.88), and 0.76 (95% CI: 0.67–0.85), respectively. Overall, these findings imply that OND exposure may exert a protective effect on patients with SAE.

**TABLE 3:** Association between treatment with ondansetron and clinical outcomes after propensity score matching using multiple regression analysis.

	HR(95%CI)	HR(95%CI)	HR(95%CI)	HR(95%CI)
	for 30-day mortality	for 90-day mortality	for 180-day mortality	for 360-day mortality
Model 1	0.71 (0.63-0.80)	0.80 (0.72-0.90)	0.83 (0.75-0.92)	0.81 (0.73-0.90)
Model 2	0.69 (0.61-0.78)	0.79 (0.71-0.89)	0.82 (0.74-0.92)	0.81 (0.73-0.90)
Model 3	0.64 (0.56-0.73)	0.75 (0.66-0.84)	0.78 (0.69-0.88)	0.76 (0.67-0.85)

Model1 was unadjusted.

Model2 was adjusted by HR, mbp; RR, temperature.

Model3 was adjusted by HR, mbp; RR, temperature age, Men, Hypertension, Diabetes, Myocardial infarct,AHF, Chronic pulmonary disease, Liver disease, Hemoglobin, Anion gap, Creatinine, Charlson comorbidity index, SAPSII,SOFA, Age score, Dexmedetomidine, Midazolam, Vasopressin.

HR, hazard ratio.

### Subgroup analysis

**FIGURE 4:**
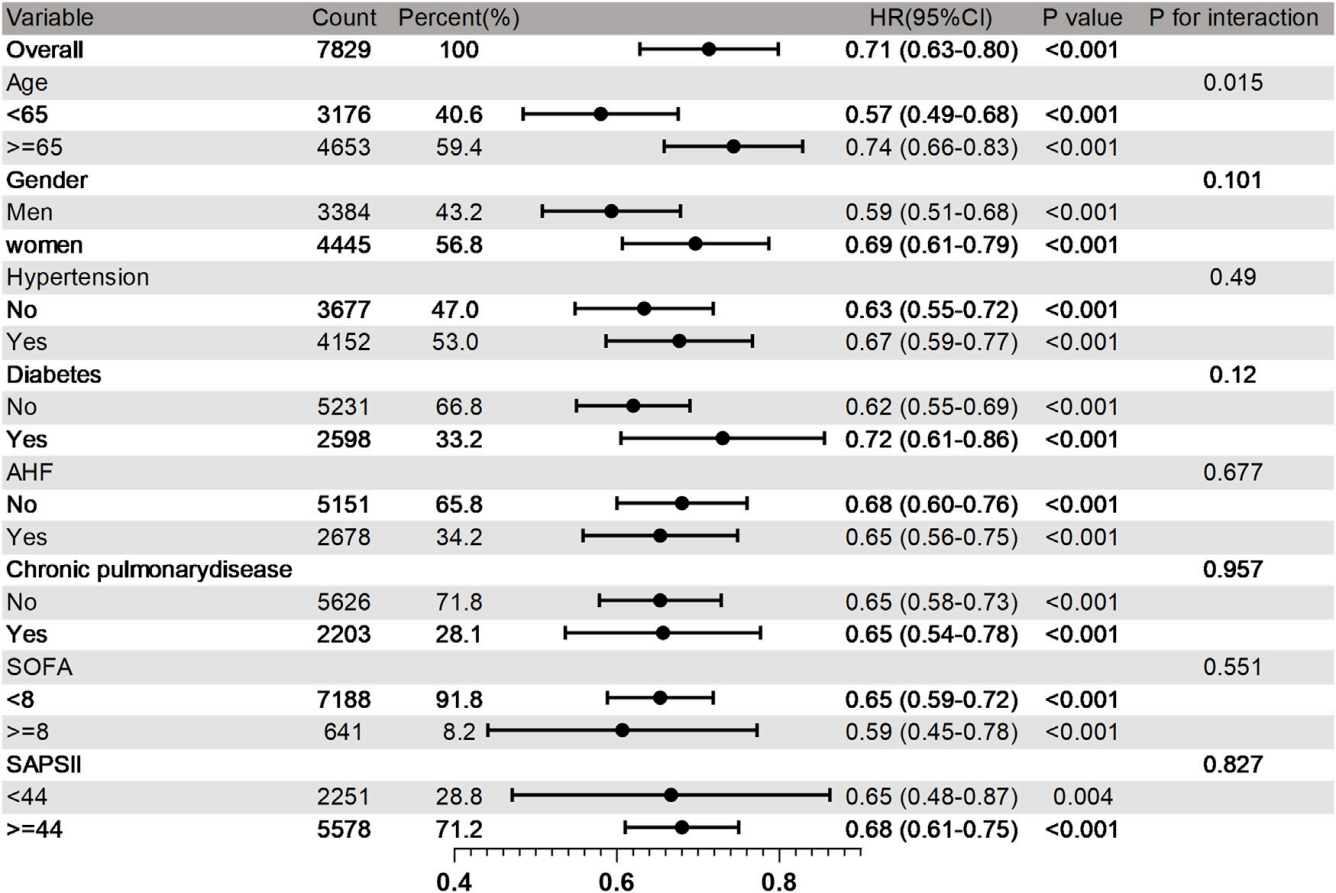
Subgroup analysis of 30 days mortality rate in SAE patients. HR, Risk Ratio; CI, Confidence Interva.

Research has revealed that OND treatment can significantly reduce the 30 - day mortality rate of SAE patients, with an HR of 0.71 (95% CI: 0.63–0.80). Subsequently, we conducted a multivariate subgroup analysis to explore how demographic data, ICU admission comorbidities, and clinical indicators influence patient prognosis. Subgroups were defined based on age, sex, hypertension, diabetes, AHF, chronic lung disease, SAPSII, and SOFA (Figure 4).

The results demonstrated that the therapeutic effect of OND on 30 - day mortality was generally consistent across most subgroups, and no significant interaction was detected (p > 0.05 for interaction). However, certain subgroups exhibited more pronounced benefits. Compared with the 30 - day mortality rate of 0.74 (95% CI: 0.66–0.83) in elderly patients aged 65 and above, the 30 - day mortality rate in adult patients under 65 was lower, at 0.57 (95% CI: 0.49–0.68). The subgroup analysis suggests that although OND use is widely beneficial for SAE patients, adult patients under the age of 65 may derive greater survival benefits from OND treatment.

### Sensitivity analysis

We calculated E-values to assess the potential influence of unmeasured confounding on the association between OND administration and mortality. The results indicate that to explain away the observed association between OND and reduced 30-day mortality, an unmeasured confounder would need to be associated with both the exposure and the outcome by a relative risk magnitude greater than 2.50. This suggests that the observed association is robust, as it is unlikely that residual confounding alone—particularly from factors stronger than known clinical risk factors—could account for the effect size. Similarly, the associations at 90 days (E-value >2.00), 180 days (E-value >2.12), and 365 days (E-value >1.88) would require substantial unmeasured confounding to be nullified (Table 4). Furthermore, a landmark sensitivity analysis conducted at 5 days after ICU admission yielded consistent results, with the survival advantage of the OND group remaining statistically significant (p < 0.001), indicating that the observed association is unlikely to be substantially biased by immortal time (Supplementary Figure S1).

**TABLE 4:** E-values associated with mortality in SAE patients treated with ondansetron after propensity score matching.

Outcome	E-value	Upper limit of 95% CI
30-day mortality	2.50	2.08
90-day mortality	2.00	1.67
180-day mortality	2.12	1.67
360-day mortality	1.88	1.53

## Discussion

This retrospective investigation scrutinized the association between the administration of OND and mortality in patients with SAE. The results following PSM demonstrated that OND usage significantly decreased mortality rates at 30, 90, 180, and 360 days. These discoveries provide evidence suggesting that OND may assume a pivotal role in the comprehensive treatment of SAE.

Although OND is predominantly used for its antiemetic properties, recent studies have suggested potential prognostic benefits for critically ill patients (Wang et al., 2024; Boshen et al., 2023; Zhou et al., 2022). A retrospective study conducted by the US Department of Veterans Affairs found that OND can reduce the 30 - day all - cause mortality rate and ICU mortality rate in hospitalized patients infected with COVID-19 (Bayat et al., 2021). Another cohort study showed that OND exposure is associated with decreased hospitalization, 28 - day, and 90 - day mortality rates in critically ill sepsis patients, indicating its potential to enhance the prognosis of sepsis patients (Yang et al., 2023). Yang et al. discovered that early use of OND is associated with a reduced risk of death from critical myocardial infarction, and its beneficial effects are mediated through anti - inflammatory actions (Boshen et al., 2023). Our research findings are in line with these previous results, suggesting that OND use can reduce both short - term and long - term mortality rates in SAE patients.

Systemic inflammation triggered by sepsis can induce SAE, with neuroinflammation and oxidative stress being crucial mechanisms underlying SAE (Li et al., 2025; Manabe and Heneka, 2022; Pan et al., 2022). As a first - line antiemetic agent, OND exerts its effects by antagonizing 5 - hydroxytryptamine 3 (5 - HT3) receptors and is widely utilized due to its outstanding efficacy and minimal side effects (Wilde and Markham, 1996). The mechanism by which OND reduces mortality in SAE patients is not fully understood. Current evidence indicates that 5 - HT3 receptors are expressed in immune cells, such as T cells and macrophages, and OND may mitigate systemic inflammation in SAE by modulating immune responses (Liu et al., 2021). A bioinformatics analysis suggested that OND reduces neutrophil extracellular trap (NET) formation by regulating key targets (tlr8, NFKB1, Mapk14, NE, and MPO). This mechanism may alleviate excessive inflammatory damage in critical illnesses (Tao et al., 2024). Another preclinical study demonstrated that OND improved renal function markers (serum creatinine and blood urea nitrogen), reduced oxidative stress (through glutathione recovery and malondialdehyde inhibition), and alleviated systemic inflammation by downregulating nuclear factor kappa B (NF - κB) and cyclooxygenase - 2 (COX - 2) (Mohamed and Shouman, 2025). These effects provide a biological foundation for investigating OND as an adjuvant therapy for SAE patients. In addition, OND is mainly metabolized in the liver. Due to the active liver function metabolism in young patients, the bioavailability of OND can be higher, and effective blood drug concentrations can be quickly achieved (Alqahtani et al., 2023). Our subgroup analysis also suggests that adult patients under the age of 65 may benefit more from OND treatment in terms of survival.

The results of this study indicate that the OND group exhibited shorter ICU stays and duration of mechanical ventilation compared to the non-OND group, a finding consistent with previous research (Tao et al., 2023). Franziska et al. believe that prolonged mechanical ventilation is a factor associated with prolonged hospitalization and increased mortality (Trudzinski et al., 2022). Therefore, we speculate that OND may alter the acute impact on SAE by reducing the need for mechanical ventilation and ICU time In clinical practice, a primary safety concern regarding OND is its potential to prolong the QT interval, which may predispose patients to life-threatening arrhythmias (Al-Ramadan et al., 2025). When screening for OND -associated arrhythmia risk, particular attention should be paid to high-risk patients and those receiving intravenous administration (Freedman et al., 2014). Notably, a report involving individuals undergoing hemodialysis suggests that OND is associated with elevated short-term cardiac risk in this specific population (Ismail et al., 2024). In contrast to that cohort, our study focused exclusively on SAE patients, and we observed no significant difference in the incidence of QT interval prolongation between the two groups. Further prospective clinical trials are warranted to validate these findings.

This observational study analyzed the association between OND use and clinical outcomes in SAE patients. Our findings offer new insights and highlight a potential opportunity for improving SAE treatment strategies. Key strengths include the utilization of a large, high-quality dataset from the MIMIC-IV database (version 3.1), which enhances the statistical power and generalizability of the results for critically ill populations. The application of PSM minimized selection bias between the OND and non-OND groups and balanced baseline characteristics, thereby strengthening the validity of the observed associations. Subgroup analyses were employed to identify specific patient subgroups that may derive the greatest survival benefit from OND. Furthermore, E-values were used to quantify the potential impact of unmeasured confounding.

However, several limitations must be acknowledged. First, the observational design inherently precludes definitive causal inferences regarding the relationship between OND use and improved survival. Although PSM was employed, residual confounding from unmeasured variables may persist. Furthermore, while the definition of OND exposure during the ICU stay could introduce immortal time bias, our landmark sensitivity analysis at 5 days yielded consistent results, strengthening the validity of the primary findings. Second, the single-center origin of the data may limit its external validity and generalizability to other settings with differing demographic or clinical profiles. Third, as SAE involves acute neurological deficits, and the MIMIC-IV database lacks comprehensive brain imaging records, we were unable to assess the neurological outcomes of OND treatment on SAE. Fourth, OND is typically administered in the ICU for postoperative or drug-induced nausea and vomiting, rather than specifically for SAE. As the MIMIC database does not precisely document reasons for medication use, indication-related confounding may remain despite adjustment for comorbidities and illness severity. Fifth, other 5-HT3 receptor antagonists (e.g., palonosetron, tropisetron) were excluded from the analysis due to their infrequent use within the MIMIC-IV database. Finally, due to the constraints of the database, we could not elucidate the specific mechanistic pathways of OND. Further basic science investigations and prospective clinical trials are necessary to confirm our findings.

## Conclusion

This study presents compelling evidence indicating that the administration of OND is correlated with a decline in mortality rates at 30, 90, 180, and 360 days in patients with SAE. Moreover, it shortens the duration of ICU stay and mechanical ventilation without substantially elevating the risk of QT interval prolongation. These results imply that OND holds promise as a therapeutic agent for enhancing the short - term and long - term prognosis of SAE patients, thereby potentially improving their clinical outcomes. Nevertheless, additional prospective studies are warranted to validate these findings.

## Data Availability

Publicly available datasets were analyzed in this study. This data can be found here: https://github.com/MIT-LCP/mimic-code/.

## Supplementary material

SUPPLEMENTARY FIGURE S1 Landmark analysis before 5 days (P < 0.001) and after 5 days (P < 0.001).

## Abbreviations

SAE, Sepsis-associated encephalopathy; OND,ondansetrn; AHF, acute heart failure; HR, heart rate; Mbp,mean blood pressure; RR, respiratory rate; Charlson, Charlson Comorbidity Index.
