# Supplementary figures and images for: The correlation between the use of ondansetron and mortality in sepsis associated encephalopathy patients: a retrospective ICU cohort study
Source: Front Pharmacol. 2025 Nov 10;16:1712328. doi: 10.3389/fphar.2025.1712328 (PMC12640904; doi:10.3389/fphar.2025.1712328)

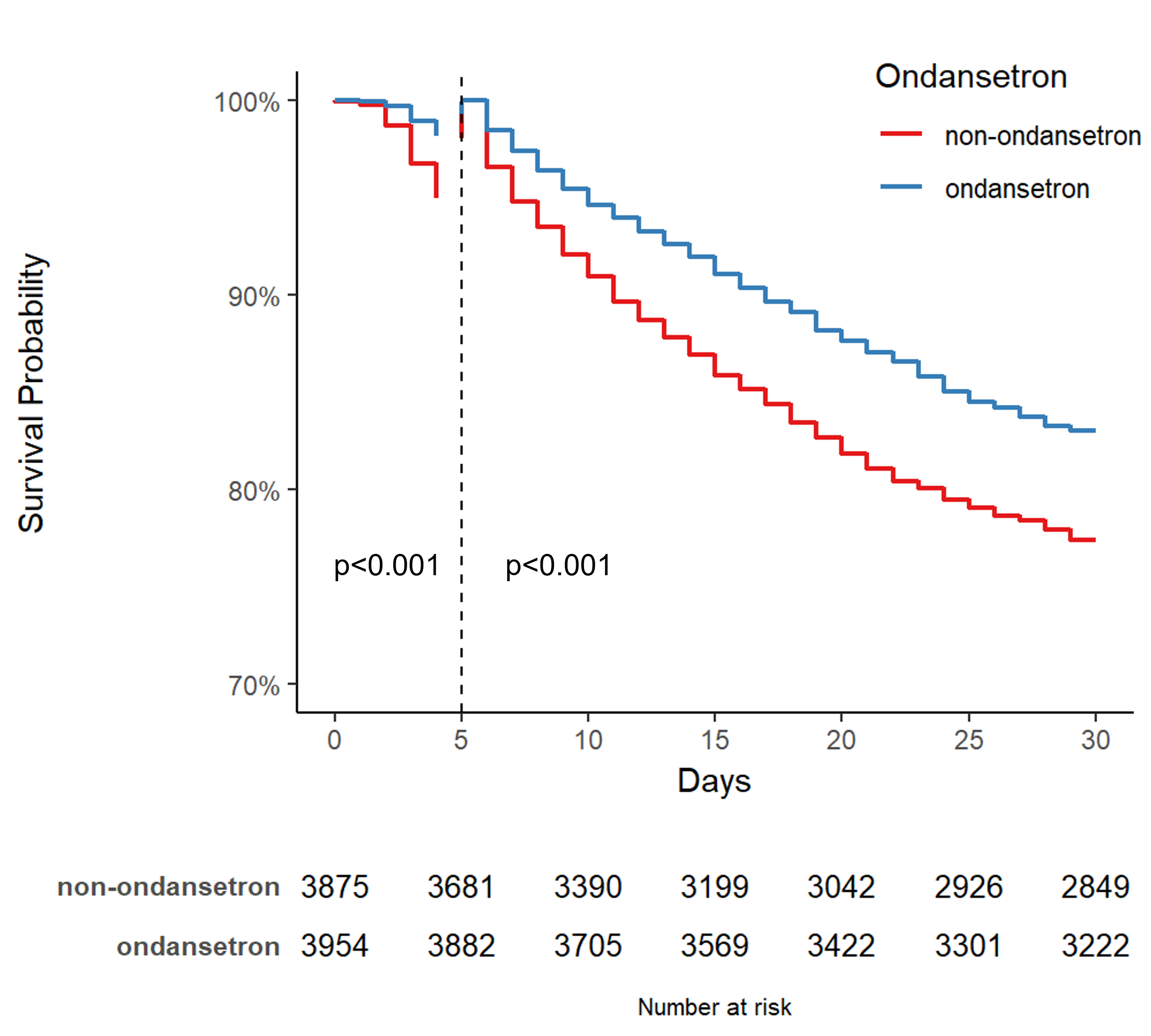

Supplement: Supplementary file 1 [file Image1.tif]
